# Oro-facial rehabilitation of cancer patients: ‘Zygomatic 2019’-1–2 March 2019, London, UK

**DOI:** 10.3332/ecancer.2019.925

**Published:** 2019-04-30

**Authors:** Paul HR Wilson, Kumni Fasanmade, Prad Anand

**Affiliations:** Department of Maxillofacial Surgery, Orthodontics and Restorative Dentistry, Oxford University Hospitals NHS Foundation Trust, Oxford OX3 9DU, UK

**Keywords:** zygoma, implant, maxilla, maxillofacial, conference, osteoradionecrosis, oral, rehabilitation

## Abstract

This first truly global conference on the use of zygomatic implants for the oral reconstruction of patients with compromised or deficient maxillae was held at the Museum of London from 1 to 2 March 2019. It attracted over 200 clinicians and academics from 24 nations and reviewed over 25 years’ experience and research in this field. This conference presented concepts heralding new opportunities for the oro-facial rehabilitation of doctor-induced osteonecrosis of the jaw (ONJ), both osteonecrosis radiation-induced (ORN) and medication-induced ONJ (MRONJ) in oncology patients.

The conference was opened by Dr Luc Vrielinck (University of Ghent, Belgium), who presented the role of zygomatic implants in a historical perspective. He detailed their development from Professor Branemark *et al*’s [1] seminal work, to where they were employed in the restoration of a fixed dentition for a patient with cleft lip and palate. Rapidly they were employed for the treatment of the edentulous atrophic maxillae, in patients who could not tolerate conventional dentures. The early surgical techniques were presented, where a machined implant was placed into the body of the zygoma, often traversing the lateral maxillary air sinus wall. The challenges of 45° abutment placement and implant angulation were discussed, and the high rates of postoperative sepsis were presented along with early attempts to combat this.

Dr Claudio Brenner from The National Cancer Institute, Chile, considered the placement of zygomatic implants in the postoperative head and neck cancer patient. He discussed the role of careful zygoma assessment via CT, to gain a quantitative and qualitative understanding of available bone for zygomatic implant stability and osseointegration. The shape of the lateral maxillary air sinus wall in relation to implant trajectory was considered along with the question of whether the lateral bone was necessary for long-term implant success.

The relationship of a zygomatic implant to the lateral sinus wall, and indeed the Schneiderian membrane itself was the topic presented in detail by Dr Carlos Aparicio (Universities of Gothenburg and Barcelona). He introduced the concept and classification system of the zygoma anatomy-guided approach (ZAGA) [2] method of treatment. This involved placing the zygomatic implant in an extra-zygomatic position so that complications can be reduced, and in most cases, avoided. Ten-year follow-up data were presented [2]. The position of the implant head is positioned more buccal in the palate, making the fixed prostheses much less bulky, which improves maintenance and oral space for speech and mastication.

Dr Ulf Nannmark (University of Gothenburg, Sweden) explored the range of biomaterials for reconstruction of the challenging edentulous maxilla, including the role of ceramic ‘membranes’ to promote filler-less osseous regeneration prior to zygomatic implant treatment. He has developed novel materials, which release bioactive molecules to upregulate bone formation, and he hoped to bring these to market.

The role of the ENT surgeon in pre-assessment of the maxillary sinuses was considered by Mr Samuel Leong of the Skull Base Team at Aintree University Hospital NHS Foundation Trust, Liverpool, UK. He recommended that pre-assessment patient questionnaires, such as the Sino-Nasal Outcome Test (SNOT-22) [3], should be employed for all patients where zygomatic implants are considered. Such indices would sift out those cases where formal nasendoscopy is required, and sino-nasal pharmacological therapy or functional endoscopic sinus surgery (FESS) is required.

Day 1 was rounded off by clinical technique presentations by Mr Guy McLellan (Charing Cross Hospital, London, UK) and Dr Andrea Tedesco (S. Chiara Hospital, University of Pisa, Italy). Mr McLellan introduced the role and methodology of the extended maxillary sinus lift approach with simultaneous zygomatic implant placement. He advocated in-fracturing the lateral maxillary sinus wall and placing osteogenic filler and an autologous fibrin membrane harvested from the patient’s blood (PRGF-Endoret). Dr Tedesco presented an alternative approach, where piezosurgical site preparation of the lateral sinus wall and zygoma was developed before implant placement. Early clinical preliminary results were promising but research was needed.

Day 2 had a distinct emphasis on prosthodontic rehabilitation of zygomatic implants. Professor Dale Howes (University of Witwatersrand, South Africa) considered the prosthodontic implications of fixture head position and the issues for both maintenance and prosthesis construction. The role of 55° abutment angulation was explored, providing screw access within the prosthodontic envelope. Removal versus fixed restoration was also considered, in terms of patient quality of life and function. Challenges to the chewing cycle of a lingualised occlusion were discussed in great depth.

Dr Luc Vrielinck (Belgium) presented a very honest and candid lecture on what he’s learned over the past 25 years in terms of complications [4]. Complications were classified and presented as clinical cases, complete with full documentation and outcomes. The most severe was the inadvertent intra-cranial implant placement, with controlled implant removal and then another zygomatic fixture of shorter length was placed. The concept of re-treatment of cases after 15 years or so was highlighted, and the fact that such reconstructions have a lifespan was discussed in a very open manner.

Professor Dale Howes maintained this theme of honesty by considering the issues with long-term prosthodontic failure and maintenance. Cement versus screw retention of prostheses was discussed, and the evidence basis presented. He advocated screw retention, given its retrievable nature and ease of repair. Poor implant head position and the effect on prosthodontic outcome over the long-term was highlighted, giving a salutary lesson to the surgeons in the audience.

Finally, treatment planning in maxillectomy patients and the role of combining zygomatic implants with soft tissue and composite grafts in oncology patients were considered by Mr Andrew Dawood (University College London and the Chelsea and Westminster Hospitals, London, UK), Mr Chris Butterworth (University Hospital Aintree, Liverpool, UK) [5] and Dr Claudio Brenner (Chile). The possibilities of zygomatic implants in oncology patients were pushing the boundaries of what is possible, and functional and aesthetic reconstructions were now a reality. The role of reconstruction in the ONJ cancer patient was also discussed, with implant placement at the time of primary surgery.

## Conclusion

Zygomatic 2019 was an enlightening and aspirational meeting, where the historical context of care was considered alongside contemporary advances. The benefits to head and neck oncology patients were immense, in their rehabilitation back into meaningful society. The role of zygomatic implants in the oro-facial rehabilitation of head and neck cancer patients was explored, and the benefits of their use in ONJ cases was highlighted, where most forms of dentoalveolar osseointegrated implants would be contraindicated. For both ORN and MRONJ cancer survivors, zygomatic implants provide a lifeline for oral functional and aesthetic rehabilitation.
